# Toward Off-Grid Photovoltaics-Driven Hydrogen Production: A Conceptual Study on Biomass-Assisted Fe^3+^/Fe^2+^ Mediated Co-Electrolysis

**DOI:** 10.3390/molecules30214188

**Published:** 2025-10-27

**Authors:** Chunhua Zhu, Jie Yao, Meng Du, Henghui Xu, Jintao Yu, Haotian Zhu, Zeyu Zhou, Jubing Zhang

**Affiliations:** 1 Guodian Environmental Protection Research Institute Co., Ltd., Nanjing 210031, China; 2 Huaneng Jiangyin Gas Turbine Cogeneration Co., Ltd., Wuxi 214400, China; 3 School of Energy and Mechanical Engineering, Nanjing Normal University, Nanjing 210042, China

**Keywords:** co-electrolysis, Fe^3+^/Fe^2+^ redox couple, Fe^2+^ regeneration, biomass

## Abstract

As a conceptual study for low-energy hydrogen production, potentially coupled with off-grid photovoltaics, this work focuses on overcoming the constraint of the oxygen evolution reaction (OER), which features a high anode potential and significant overpotential. To reduce energy consumption, the Fe^2+^ oxidation reaction is employed to replace OER, coupled with Fe^2+^ regeneration using natural biomass. Experimental results reveal that Fe^2+^ oxidation reaction is an effective substitute, with an initial oxidation potential of 0.5 V (vs. Hg/Hg_2_SO_4_), much lower than that of OER. Fe^2+^ regeneration is notably influenced by both biomass type and reaction temperature. Chlorella pyrenoidosa (CP) achieves the highest Fe^3+^ reduction rate of 90.5% at 190 °C. Water-soluble organic compounds generated during biomass oxidation exert a negative impact on Fe^2+^ electrooxidation by accumulating on or coating the electrode surface, and the compounds derived from CP exert a less detrimental effect. Moreover, enhancing magnetic stirring, elevating temperature, and selecting an appropriate anode material can significantly boost the oxidation reaction. Under optimized conditions, the current density during electrolysis of CP filtrate at 1.1 V reaches 280 mA/cm^2^, much higher than values reported in similar studies. This highlights the great potential of this co-electrolysis approach for efficient hydrogen production driven by off-grid photovoltaic power.

## 1. Introduction

With the acceleration of industrialization and urbanization, China’s energy consumption has gradually increased in recent years [1]. Currently, fossil fuels meet the vast majority of energy demand; however, massive carbon dioxide emissions into the atmosphere have a significant impact on the environment and human health [2]. Thus, large-scale development and utilization of renewable energy sources have become increasingly urgent [3]. Solar energy is recognized as a promising solution due to its abundant availability, wide distribution and environmental friendliness [4]. The radiant energy of sunlight can be directly converted into electrical energy via the photovoltaic (PV) effect, the most widely used solar energy utilization technology to date. Consequently, China’s installed solar power capacity has shown robust growth in recent years [5]. Nevertheless, light intensity and light duration are susceptible to season, climate, weather and other factors, leading to significant fluctuations in the output power of PV systems. To ensure the safety and stability of the power grid, large-capacity energy storage devices are essential. Unfortunately, meeting this demand remains challenging at present [6].

Hydrogen is another attractive energy carrier for the future, characterized by high energy density and zero carbon emissions [7]. Industrial hydrogen production technologies primarily include fossil fuel-based routes such as coal gasification [8] and methane reforming [9,10]. These routes currently dominate the market but generate substantial greenhouse gas emissions [11]. An alternative fossil-based approach is the purification of industrial by-products (e.g., from the chlor-alkali or steel industry), which offers a transitional pathway for hydrogen production [12]. However, the fluctuating composition and complex impurities of the feedstock gas result in high purification costs. Hydrogen carrier decomposition is a highly promising hydrogen production technology, among which ammonia [13] and formic acid [14] are the most common hydrogen carriers. This technology enables the safe and efficient storage and transport of hydrogen, but the decomposition process is often energy-intensive and may generate carbon-containing byproducts. Photocatalytic water splitting directly utilizes solar energy to produce hydrogen from water using semiconductor catalysts, with recent advances focusing on low-cost and stable materials [15]. Water electrolysis is a highly environmentally friendly and mature technology, as its only inputs are water and electricity, and its only decomposition products are high-purity hydrogen and oxygen. Despite its high energy consumption, water electrolysis offers distinct advantages for large-scale applications: it provides exceptionally high-purity hydrogen, is highly scalable, and most importantly, can be directly and efficiently coupled with intermittent renewable power sources like PV to produce truly green hydrogen [16,17]. The integration of PV power and water electrolysis can simultaneously produce abundant green hydrogen and provide large-scale energy storage for fluctuating power, attracting widespread global attention [18,19]. However, the high energy consumption of water electrolysis (up to 4.5~5.0 kWh/Nm^3^ H_2_) makes this integrated energy system economically uncompetitive [20].

The reversible voltage (*E*^0^_rev_) for conventional water electrolysis is calculated as 1.23 V according to Equation (1) [21]:

*E*^0^_rev_ = −ΔG^0^/(*n*F) (1)

where ΔG^0^ denotes the standard Gibbs free energy change of the reaction, while *n* and F represent the number of electrons transferred and Faraday’s constant, respectively.

In acidic electrolytes, the oxygen evolution reaction (OER) and hydrogen evolution reaction (HER) are expressed by the following equations, with the standard equilibrium electrode potentials (at 25 °C and 1 atm) of 1.23 V and 0 V, respectively [22]:

OER: H_2_O = 2H^+^ + 0.5O_2_ + 2e^−^ *E*_a_^θ^ = 1.23 V (2)

HER: 2H^+^ + 2e^−^ = H_2_ *E*_c_^θ^ = 0.00 V (3)

In practical water electrolysis for hydrogen production, the operating voltage is much higher than 1.23 V to achieve a considerable hydrogen yield, due to various additional overpotentials [23]. Approximately 40% of potential losses are attributed to OER, regarded as the primary cause of the high operating voltage [24]. Currently, the main strategies to reduce energy consumption in water splitting include synthesizing highly active OER catalysts and optimizing water electrolysis systems [25]. Although high-performance anode catalysts can reduce OER overpotential, the operating voltage of water electrolysis cannot break through the limitation of the reversible potential (1.23 V) [26]. Thus, replacing the low-activity OER with the oxidation of other easily oxidizable substances has been proposed [27]. Additionally, proton-dependent redox mediators, such as Fe^3+^/Fe^2+^ [28], Fe(CN)_6_^3−^/Fe(CN)_6_^4−^ [29], POM(Ox)/POM(Red) [30], and vanadium species, have been considered to be reliable alternatives [31,32]. Among common redox pairs, the Fe^3+^/Fe^2+^ redox couple has been widely studied owing to its suitable electrode potential (0.77 V), high solubility in acidic solutions, low toxicity, and low cost [33]. Fe^3+^ generated via electrooxidation can be easily reduced to Fe^2+^ by coal, biomass, organic waste, etc. [34]. This regeneration process produces a certain amount of water-soluble organic compounds (WSOCs), some of which may affect Fe^2+^ electrooxidation. However, related research in this area is rarely reported. In this study, four types of natural biomass were used as reductants for Fe^2+^ regeneration, and hydrogen was produced at low operation voltages by replacing OER with the Fe^2+^ oxidation reaction.

## 2. Results and Discussion

### 2.1. Electrooxidation of Fe^2+^

The primary focus of this study is biomass-assisted Fe^3+^/Fe^2+^ mediated co-electrolysis for hydrogen production, making hydrogen yield a key technical parameter. Given the extremely low actual hydrogen production in the experiments, online measurement of hydrogen volume poses significant challenges. Since hydrogen production is directly correlated with the number of electrons, it can be indirectly reflected by current magnitude. Therefore, accurate estimation of Faradaic efficiency becomes particularly important.

**Figure 1:**
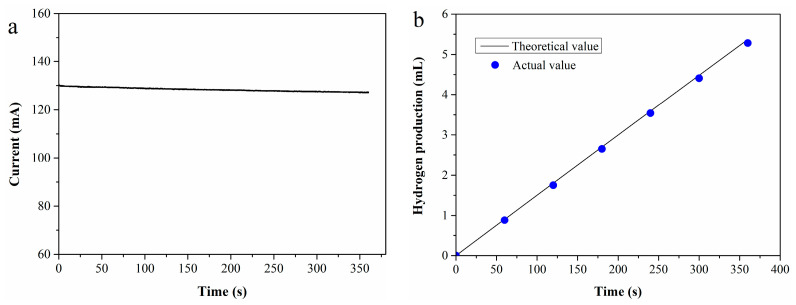
(**a**) *I*-*t* curve of 0.4 M Fe^2+^ solution; (**b**) theoretical vs. experimental hydrogen production.

To evaluate the Faraday efficiency, an *i*-*t* test was performed at 70 °C using a 0.4 M Fe^2+^ standard solution as the anolyte, with the resulting *i*-*t* curve presented in Figure 1a. Based on the test data, the theoretical hydrogen production *V*_0_ (mL) can be calculated using Equation (4) [35]:

(4) $$V_{0}=1000\times 22.4\int_{0}^{t}i\left(t\right)dt/\left(nF\right)$$

where *i*(t) denotes the current value from the *i*-*t* curve, *n* (*n* = 2) represents the number of electrons transferred, and F is Faraday’s constant.

The actual hydrogen production (*V*_1_) was determined by water displacement collection, with Faraday efficiency defined as the ratio of *V*_1_ to *V*_0_. Figure 1b shows the comparison between theoretical and actual hydrogen production across different durations. As observed in the figure, the actual hydrogen production closely approximates the theoretical value, resulting in a high Faraday efficiency of 98.5%.

**Figure 2:**
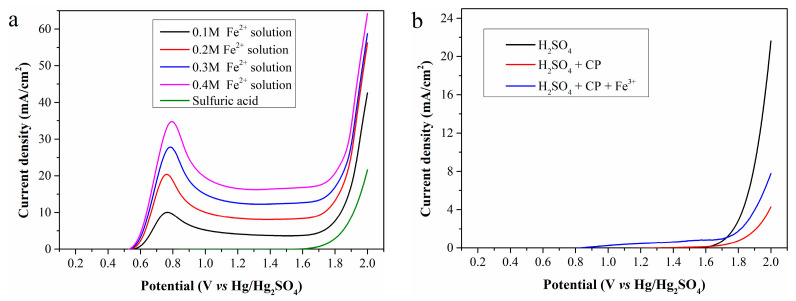
LSV curves of (**a**) the standard Fe^2+^ solutions and (**b**) the mixture of CP and Fe^3+^.

Using the standard Fe^2+^ solution (Fe^2+^ concentration: 0.1, 0.2, 0.3 and 0.4 mol/L, respectively) and 0.5 mol/L sulfuric acid as the anolyte, the LSV curves obtained with a scan rate of 10 mV/s and an anode potential range of 0.2–2.0 V (vs. Hg/Hg_2_SO_4_) is shown in Figure 2a.

As shown in the figure, no current is generated during electrolysis of the sulfuric acid even when the anode potential increases to 1.6 V, mainly due to high anodic overpotential. Thus, OER is ignored when the anode potential is below 1.6 V in subsequent studies. For electrolysis of the standard Fe^2+^ solution, oxidation current can be detected when the anode potential exceeds 0.5 V, attributed to Fe^2+^ oxidation. Therefore, Fe^2+^ electrooxidation behavior is discussed only within the anode potential range of 0.5–1.6 V. As the anode potential increased, the current density first rises sharply, reaches a maximum at about 0.8 V, and then slowly stabilizes. Furthermore, a high Fe^2+^ concentration in the anolyte significantly improves the current density, ultimately enhancing hydrogen production efficiency.

According to Figure 2b, electrolysis of sulfuric acid with 5 g of CP added to the anode chamber still generates no current at the anode potentials below 1.6 V. Unfortunately, the presence of CP even reduces the current density at high anode potential. However, when 0.02 mol of iron (III) sulfate is further added to the anode chamber containing CP, a small current is detected at low anode potentials, caused by electrooxidation of regenerated Fe^2+^. In general, CP cannot be directly oxidized on the Pt electrode within the selected anode potential range but can be indirectly oxidized via Fe^2+^ regeneration. The low current density indicates a low Fe^2+^ concentration in the anolyte, further reflecting a low Fe^3+^ reduction rate at room temperature.

### 2.2. Regeneration of Fe^2+^

**Figure 3:**
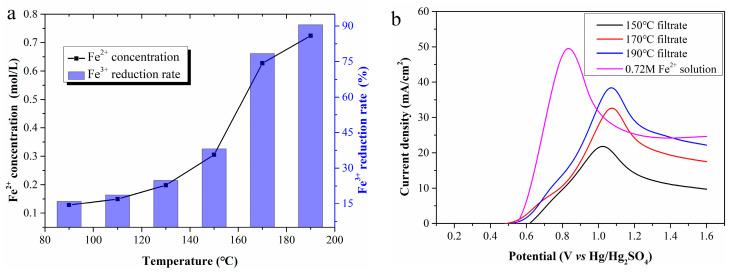
(**a**) The Fe^2+^ concentration in the filtrates; (**b**) LSV curves of the filtrates.

Reaction temperature is the most critical factor in Fe^2+^ regeneration. Figure 3a shows Fe^2+^ concentrations in filtrates prepared at 90, 110, 130, 150, 170, and 190 °C, estimated as 0.13, 0.15, 0.20, 0.31, 0.63, and 0.72 mol/L, respectively. High reaction temperatures clearly accelerate Fe^3+^ reduction, ultimately increasing the cumulative Fe^2+^ amount in the filtrate. The Fe^3+^ reduction rate in the filtrate prepared at 190 °C reaches 90.5%, much higher than that at 170 °C. It can be inferred that a near-100% reduction rate might be achieved at higher temperatures. However, temperatures above 200 °C were not tested due to limitations of the reactor lining material.

Figure 3b presents LSV curves using filtrates prepared at different temperatures as the anolyte. As Fe^2+^ concentration increases, the current density rises greatly, and the anode potential corresponding to the peak current density also presents an upward trend. Compared with the LSV curves in Figure 2a, the peak current density potential shifts to approximately 1.05 V, with a continuous decrease in current density after reaching the maximum. To clarify this, a 0.72 mol/L standard Fe^2+^ solution was electrolyzed, and its LSV curve was compared with that of the 190 °C filtrate. Despite identical Fe^2+^ concentration, the peak current density of the former is significantly lower than that of the latter, and the corresponding anode potential is approximately 0.2 V higher. These differences are initially attributed to WSOCs in the anolyte formed by the CP oxidation.

Since current density largely depends on Fe^2+^ concentration, enhancing the Fe^3+^ reduction rate is crucial for improving hydrogen production efficiency. Table 1 summarizes cumulative Fe^2+^ concentrations in filtrates after reducing 0.4 mol/L iron (III) sulfate solution (Fe^3+^ concentration of 0.8 mol/L) with CP, RS, PS, and BD at 190 °C.

It can be seen from Table 1 that Fe^2+^ concentration is highly dependent on biomass type. CP achieves a high reduction rate of 90.5%, while RS reduces only 75.4% of Fe^3+^. CP contains relatively high levels of proteins and lipids, which provide abundant dissociable active groups (e.g., sulfhydryl and carboxyl groups) that readily release electrons, endowing CP with strong reducibility. Although lignin typically exhibits a stronger reducing ability than proteins and lipids. Since RS contains approximately 15% lignin, it should have achieved a higher Fe^3+^ reduction rate. However, the lignin structure in RS contains more p-hydroxyphenylpropane units, and the unit has relatively high stability and generally does not participate in reduction reactions, resulting in poor reduction ability of RS.

**Figure 4:**
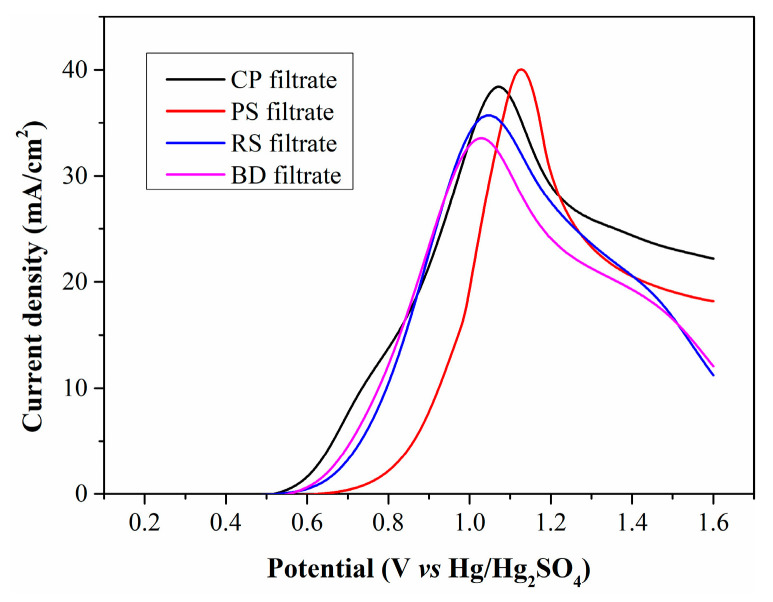
LSV curves of filtrates produced from different types of biomass.

Figure 4 illustrates the LSV curves of the four filtrates. All curves exhibit a similar profile and an almost identical initial oxidation potential of 0.5 V (vs. Hg/Hg_2_SO_4_), which is attributed to the oxidation of Fe^2+^. The peak current densities for the filtrates produced by CP, RS, PS, and BD are calculated as 38.4, 35.6, 39.8 and 33.6 mA/cm^2^, respectively. The filtrates from CP and PS have higher Fe^2+^ concentrations than those from RS and BD, generating larger current densities. However, the PS filtrate exhibits significantly lower current density in the low anode potential range, suggesting that WSOCs formed by PS oxidation exert a more negative impact on Fe^2+^ electrooxidation.

**Figure 5:**
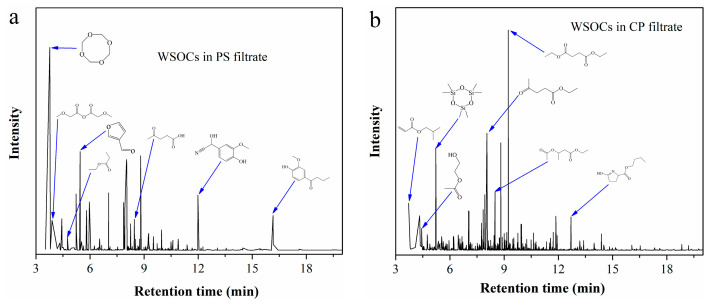
Total ion chromatogram of the extracts from (**a**) PS filtrate; (**b**) CP filtrate.

WSOCs in the filtrates from CP and PS were extracted with ethyl acetate, and the resulting extracts were analyzed by GC-MS. The total ion chromatogram in Figure 5 reveals that the typical components with high relative peak area in CP filtrate include diethyl succinate, ethyl levulinate, isobutyl acrylate, and ethyl 2-hydroxypropionate. In contrast, PS filtrate primarily contains levulinic acid, ethyl propionate, methoxyacetic anhydride, and 3-furaldehyde.

**Figure 6:**
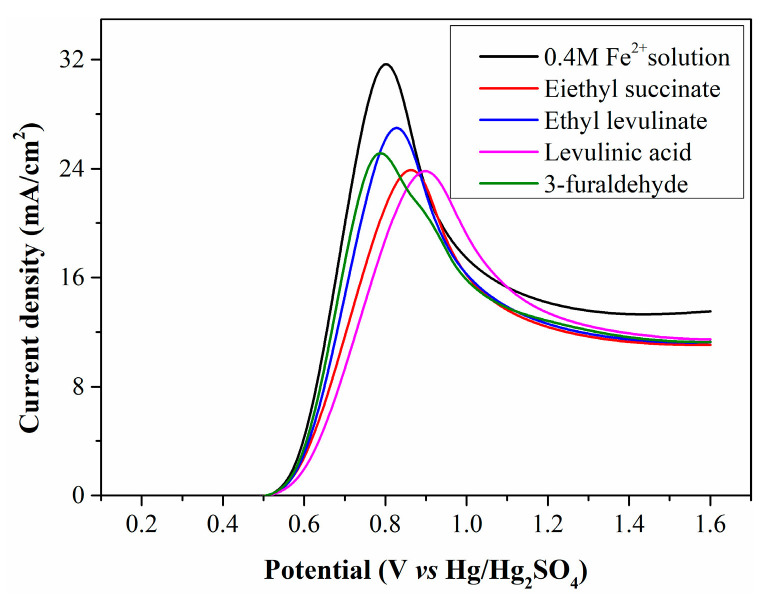
LSV curves of simulated solutions containing typical organic substances.

To confirm whether Fe^2+^ electrooxidation is restricted by WSOCs, simulated solutions were prepared by adding diethyl succinate, ethyl levulinate, levulinic acid, and 3-furaldehyde to a 0.4 mol/L standard Fe^2+^ solution, then electrolyzed in the anode chamber. The resulting LSV curves (Figure 6) show that the initial oxidation potential of the simulated solutions matches that of the standard Fe^2+^ solution, indicating these organic substances are not oxidized at low potentials. However, the peak current density of the simulated solutions is significantly lower than that of the standard Fe^2+^ solution, confirming the harmful effect of WSOCs on Fe^2+^ electrooxidation. Some organic substances exhibit high surface activity, reducing solution surface tension and facilitating their accumulation on the electrode surface. This accumulation decreases contact efficiency between Fe^2+^ and the anode surface, lowering current density.

**Figure 7:**
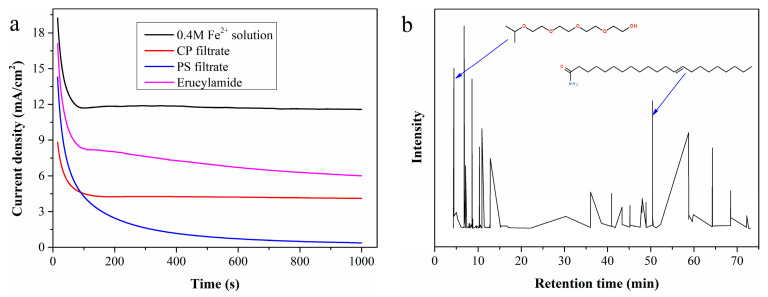
(**a**) *I*-*t* curves of simulated solutions containing typical organic substances; (**b**) total ion chromatogram of the viscous organic substances coated on the electrode surface.

Figure 7a shows *i*-*t* measurement results using CP and PS filtrates as the anolyte. The current density of CP filtrate initially drops drastically and then stabilizes at a constant value. The sharp decrease in current density may be attributed to the rapid accumulation of organic substances on the electrode surface, while the subsequent constant current density indicates that the hydrogen production system can operate stably. In contrast, the current density of the PS filtrate continues to decrease throughout the *i*-*t* test, dropping almost to zero after 1000 s of continuous testing. Post-test observations revealed a thin layer of viscous organic substances adhering to the electrode surface when using PS filtrate as the anolyte, whereas this phenomenon did not occur when electrolyzing CP filtrate. Whether these viscous coatings cause the continuous decrease in current density requires further confirmation.

The viscous organic substances coated on the electrode surface were dissolved with ethyl acetate, and the resulting solution was analyzed by GC-MS. According to the total ion chromatogram in Figure 7b, 3,6,9,12-tetraoxatetradecan-1-ol and erucylamide are easily identified as key components. A simulated solution prepared by adding 5 mL of erucylamide to 95 mL of 0.4 mol/L standard Fe^2+^ solution was electrolyzed, with the LSV curve (Figure 7a) confirming that erucylamide reduces the Fe^2+^ electrooxidation rate. Additionally, the clean surface was recoated with erucylamide after electrolysis of the simulated solution.

### 2.3. Optimization of Experimental Conditions

**Figure 8:**
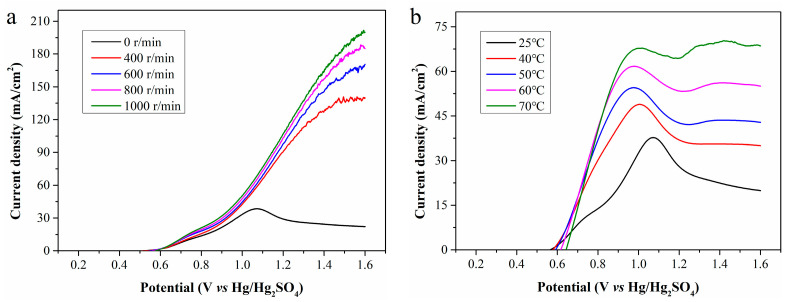
LSV curves of CP filtrate at (**a**) different rotation speeds; and (**b**) different temperatures.

LSV curves of the standard Fe^2+^ solutions and the filtrates exhibit significant oxidation peaks, indicating the electrochemical reaction is severely constrained by the diffusion of Fe^2+^ to the electrode surface. To enhance the mass transfer of Fe^2+^, magnetic stirring was implemented during electrolysis, and the LSV curves obtained at rotation speeds of 400, 600, 800, and 1000 r/min are presented in Figure 8a. As the rotation speed increases, the current density rises rapidly, and the oxidation peak disappears. At the anode potential of 1.6 V, the maximum current densities at 400, 600, 800, and 1000 r/min are 140.7, 170.5, 188.1, and 201.5 mA/cm^2^, which are significantly higher than the initial value of 38.4 mA/cm^2^. Magnetic stirring enhances Fe^2+^ diffusion from the bulk solution to the electrode surface and Fe^3+^ diffusion in the reverse direction, greatly increasing electrooxidation rate and current density. Strong agitation also partially detaches WSOCs previously accumulated on the anode surface, enabling more sufficient contact between Fe^2+^ and the anode and further increasing current density. Higher rotation speed reduces ion diffusion resistance and accelerates chemical reaction rate. However, when the mass transfer resistance is sufficiently low, the reaction rate is no longer limited by mass transfer, and excessively high rotation speed will deteriorate the contact between the electrode and electrolyte.

Besides mass transfer, reaction temperature is another key parameter affecting the electrooxidation of Fe^2+^. As is shown in Figure 8b, electrolysis conducted at different temperatures without magnetic stirring reveals that increasing temperature enormously enhances the current density and causes the peak current density to shift toward the lower potential region. In other words, a higher temperature enables the electrolysis system to achieve a higher current density at a lower anode potential. At temperatures of 25, 40, 50, 60, and 70 °C, the maximum achievable current densities are 37.8, 48.9, 54.5, 61.7, and 70.3 mA/cm^2^, respectively. Although increasing temperature does not directly reduce the reaction activation energy, it endows molecules have higher energy, which is equivalent to lowering the relative height of the activation energy barrier. This allows more molecules to more easily cross the energy barrier, facilitating reaction progression and accelerating the electrochemical reaction rate. Furthermore, high temperature increases the kinetic energy of ions, promotes ion mobility in the electrolyte, and thereby improves the conductivity of the electrolyte.

**Figure 9:**
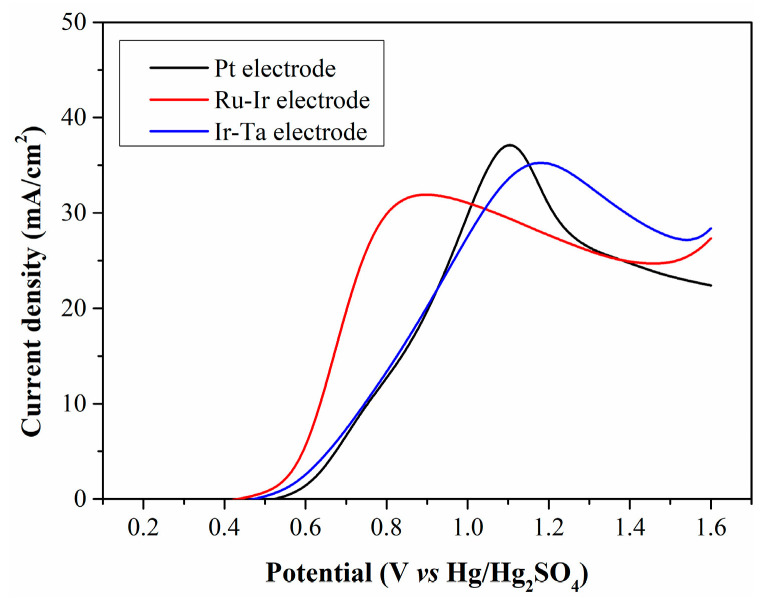
LSV curves of CP filtrate electrolyzed with different anode electrodes.

The type of anode material plays a crucial role in reducing anodic overpotential. The oxidation characteristics of Fe^2+^ on common precious metal electrodes are discussed, and the results are demonstrated in Figure 9. When the filtrate is electrolyzed using Pt and Ir-Ta electrodes, there is no significant difference in peak current density, and the LSV curves exhibit a high degree of overlap at low potentials. In contrast, the peak current density obtained with the Ru-Ir electrode is relatively low, while this electrode enables a higher current density at low potentials.

**Figure 10:**
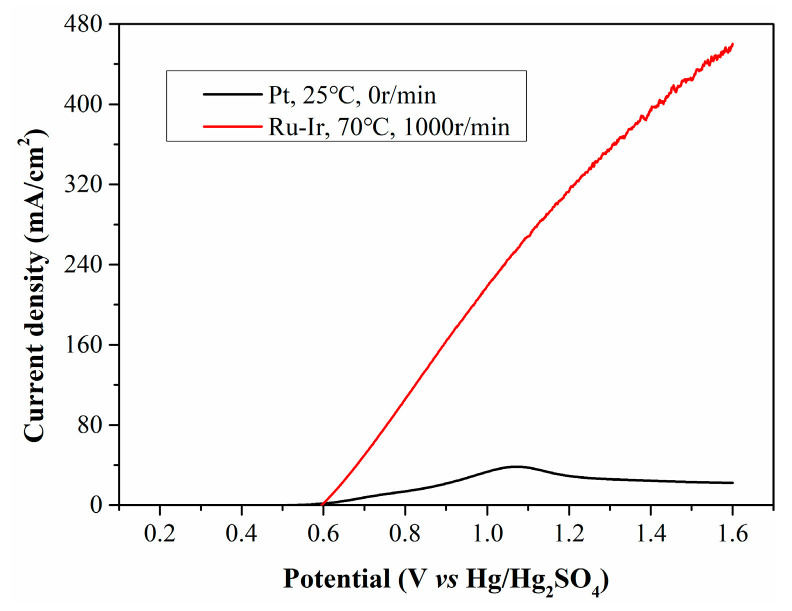
LSV curves of CP filtrate electrolyzed at opimized conditions.

With a rotation speed of 1000 r/min and a temperature of 70 °C, the filtrate from CP was electrolyzed using a Ru-Ir electrode, and the resulting LSV curve is presented in Figure 10. Under the optimized conditions, the current density increases enormously, reaching up to 280 mA/cm^2^ at 1.1 V. To compare the current density of our process with literature-reported values, three types of biomass (cornstalk, glucose and starch) reported in literature for hydrogen evolution are shown in Table 2. It can be seen that using the method proposed by us, a relatively high current density can be directly obtained with raw biomass as the reductant, which highlights that this hydrogen production system has certain advantages in terms of hydrogen production cost.

## 3. Experimental

### 3.1. Chemicals and Materials

Iron (II) sulfate (FeSO_4_·7H_2_O, 99.95%) and iron (III) sulfate (Fe_2_(SO_4_)_3_·xH_2_O, 99.95%) were used as sources of Fe^2+^ and Fe^3+^, respectively, both purchased from Shanghai Aladdin Biochemical Technology Co., Ltd. (Shanghai, China). Cerium (IV) sulfate (Ce(SO_4_)_2_, 99.9%, Shanghai Aladdin Biochemical Technology Co., Ltd., Shanghai, China) was employed to determine the Fe^2+^ concentration in solution. Sulfuric acid (98%, GR, Sinopharm Chemical Reagent Co., Ltd., Shanghai, China) and deionized water (18.2 MΩ·cm) were used for aqueous solution preparation. Four types of biomass, including chlorella pyrenoidosa (CP), rice straw (RS), pine sawdust (PS) and bean dregs (BD), were washed with 0.5 mol/L sulfuric acid and then used as reductants for Fe^2+^ regeneration.

### 3.2. Fe^2+^ Regeneration Process

For Fe^2+^ regeneration, 5 g of biomass and 0.04 mol of iron (III) sulfate were added into 100 mL of 0.5 mol/L sulfuric acid. The mixture was thoroughly stirred and transferred to a high-pressure reactor that was heated to a preset temperature and maintained at that temperature for 6 h. After reaction, the reactor was naturally cooled to room temperature, and the product was filtered to produce the primary filtrate. The obtained filter residue was fully washed with 100 mL of deionized water, followed by filtration again to obtain the secondary filtrate. The secondary filtrate was combined with the primary filtrate, and the mixture was evaporated and concentrated to 100 mL in a water bath. Finally, the biomass-regenerated filtrate was obtained. Fe^2+^ concentration in the filtrate was measured via redox titration using an automatic potentiometric titrator (ZDJ-4B, Yidian Scientific Instrument, Shanghai, China).

### 3.3. Characterization Analysis

The chromatographic analysis (GC) was conducted using an Agilent 5977A series GC/MSD system (Agilent Technologies, Santa Clara, CA, USA). Separation was achieved on an HP-5MS capillary column. The oven temperature was programmed as follows: initial temperature of 60 °C (held for 1 min), increased to 300 °C at a rate of 10 °C/min, and finally held at 300 °C for 5 min. High-purity helium (99.999%, Nanjing Shangyuan Industrial Gases, Nanjing, China) was used as the carrier gas at a constant flow rate of 1.0 mL/min. The sample (1 μL) was injected in the splitless mode. The injector temperature was set at 280 °C. The mass spectrometer (MS) was operated in the electron ionization (EI) mode at 70 eV. The temperatures of the ion source and the transfer line were set at 230 °C and 280 °C, respectively. Data were acquired in the full scan mode over a mass range of *m*/*z* 50–550.

### 3.4. Electrolysis Experiments

Electrolysis experiments were carried out in an H-type electrolyzer (Gaossunion, Tianjin, China) coupled with an electrochemical workstation (CHI660E, Chinstruments, Shanghai, China). The anode chamber contained a platinum (Pt) sheet electrode (2 cm × 2 cm) immersed in either a standard Fe^2+^ solution (0.5 mol/L sulfuric acid with a specific Fe^2+^ concentration) or the filtrate as the anolyte. The cathode chamber contained another Pt sheet electrode of the same size immersed in 0.5 mol/L sulfuric acid. The two chambers were separated by a Nafion 115 membrane to prevent iron ions from migrating to the cathode chamber. A Hg/Hg_2_SO_4_ electrode in saturated K_2_SO_4_ solution served as the reference electrode. In the electrolysis tests, the volume of electrolyte in both chambers was 100 mL. Linear sweep voltammetry (LSV) was performed at a scan rate of 10 mV/s. Amperometric *i*-*t* curve measurements were conducted at 0.8 V (vs. Hg/Hg_2_SO_4_) and terminated after 1000 s. The Nafion 115 membrane was pretreated by immersion in a mixed solution of 0.5 mol/L sulfuric acid and 3%wt H_2_O_2_ at 80 °C for 30 min, followed by washing and soaking in deionized water. All glassware was cleaned with 0.5 mol/L sulfuric acid and rinsed three times with deionized water before use.

## 4. Conclusions

This study explored hydrogen production via co-electrolysis of biomass and water, focusing on replacing OER with Fe^2+^ oxidation reaction to reduce energy consumption. Fe^2+^ oxidation reaction, with an initial oxidation potential of 0.5 V (vs. Hg/Hg_2_SO_4_), can effectively replace OER (1.6 V), enabling hydrogen production at a lower voltage and thus enhancing economic viability when coupled with off-grid PV power. Biomass-driven Fe^2+^ regeneration is critical, with temperature and biomass type being the key influencing factors. Higher temperatures promote Fe^3+^ reduction; among the tested biomass types, CP achieves the highest rate (90.5%) due to the rich active groups in its proteins and lipids, whereas RS (75.4%) is limited by stable lignin structures. WSOCs derived from biomass oxidation hinder Fe^2+^ electrooxidation through accumulation on the electrode surface. PS-derived WSOCs are most detrimental, causing near-zero current density after 1000 s of testing due to viscous coatings. Magnetic stirring enhances mass transfer; elevated temperature increases current density; and the Ru-Ir electrode outperforms Pt or Ir-Ta at low potentials. Under the optimal conditions (1000 r/min, 70 °C, Ru-Ir electrode, CP filtrate), current density reaches 280 mA/cm^2^ at anode potential of 1.1 V, which is much higher than values reported in similar studies, demonstrating the great potential of this co-electrolysis approach for efficient hydrogen production driven by off-grid PV power.

## Figures and Tables

**Table 1:** Fe^2+^ concentration in the filtrate reduced by different types of biomass.

Biomass Type	CP	PS	RS	BD
Fe^2+^ concentration (mol/L)	0.724	0.711	0.603	0.629
Fe^3+^ reduction rate (%)	90.5	88.9	75.4	78.6

**Table 2:** Comparison of different reductants for hydrogen evolution.

Electrolyser	Working Electrode	Redox Couple	Reductant	Electrolysis Temperature	Current Density	Applied Voltage	Ref.
PEMEC	graphite	Fe^3+^/Fe^2+^	Glucose	90 °C	200 mA/cm^2^	1.0 V	[35]
H type cell	graphite	Fe^3+^/Fe^2+^	Cornstalk	80 °C	22 mA/cm^2^	1.2 V vs. RHE	[36]
PEMEC	graphite	Fe^3+^/Fe^2+^	Starch	80 °C	14.4 mA/cm^2^	1.2 V	[37]
H type cell	Ru-Ir	Fe^3+^/Fe^2+^	Chlorella pyrenoidosa	70 °C	280 mA/cm^2^	1.1 V vs. Hg/Hg_2_SO_4_	This work

## Data Availability

The data presented in this study are available on request from the corresponding author.

## Abbreviations

The following abbreviations are used in this manuscript:

OER	oxygen evolution reaction
HER	hydrogen evolution reaction
PV	photovoltaic
WSOCs	water-soluble organic compounds
CP	chlorella pyrenoidosa
RS	rice straw
PS	pine sawdust
BD	bean dregs
GC-MS	gas chromatography-mass spectrometry
LSV	Linear sweep voltammetry
